# Deaths from cardiovascular disease involving anticoagulants: a systematic synthesis of coroners’ case reports

**DOI:** 10.3399/BJGPO.2021.0150

**Published:** 2021-12-15

**Authors:** Ali Anis, Carl Heneghan, Jeffrey K Aronson, Nicholas J DeVito, Georgia C Richards

**Affiliations:** 1 Oxford University Medical School, University of Oxford, Oxford, UK; 2 Centre for Evidence-Based Medicine, Nuffield Department of Primary Care Health Sciences, University of Oxford, Oxford, UK; 3 Global Centre on Healthcare and Urbanisation, Kellogg College, University of Oxford, Oxford, UK; 4 EBM Datalab, Nuffield Department of Primary Care Health Sciences, University of Oxford, Oxford, UK

**Keywords:** cardiovascular diseases, anticoagulants, inappropriate prescribing, medication errors, mortality, premature, coroners and medical examiners

## Abstract

**Background:**

The global burden of cardiovascular disease (CVD) is forecast to increase, and anticoagulants will remain important medicines for its management. Coroners' Prevention of Future Death reports (PFDs) provide valuable insights that may enable safer and more effective use of these agents.

**Aim:**

To identify CVD-related PFDs involving anticoagulants.

**Design & setting:**

Case series of coronial reports in England and Wales between 2013 and 2019.

**Method:**

A total of 3037 PFDs were screened for eligibility. PFDs were included where CVD and an anticoagulant caused or contributed to the death. Included cases were descriptively analysed and content analysis was used to assess concerns raised by coroners and who had responded to them.

**Results:**

The study identified 113 CVD-related PFDs involving anticoagulants. Warfarin (36%, *n* = 41), enoxaparin (11%, *n* = 12), and rivaroxaban (11%, *n* = 12) were the most common anticoagulants reported. Concerns most frequently raised by coroners included poor systems (31%), poor communication (25%), and failures to keep accurate medical records (25%). These concerns were most often directed to NHS trusts (29%), hospitals (10%), and general practices (8%). Nearly two-thirds (60%) of PFDs had not received responses from such organisations, which are mandatory under regulation 28 of the Coroners' (Investigations) Regulations 2013. A publicly available tool has been created by the authors (https://preventabledeathstracker.net), which displays coroners’ reports in England and Wales to streamline access, and identify important lessons to prevent future deaths.

**Conclusion:**

National organisations, healthcare professionals, and prescribers should take actions to address the concerns of coroners in PFDs to improve the safe use of anticoagulants in patients with CVD.

## How this fits in

A previous assessment of 500 PFDs identified anticoagulants as the class of drugs most often involved in fatal medication errors. This study uses innovative methods to automatically collect all available PFDs between 2013 and 2019 to identify deaths from CVD when the use of or lack of anticoagulants caused or contributed to the death. Coroners raised hundreds of concerns in their reports, including issues with communication, following protocols, education and training, access to resources, and safety. Despite repeat concerns with national relevance being identified, most CVD-anticoagulant PFDs were sent locally to NHS trusts, hospitals, and general practices, limiting their ability to reduce harms and prevent premature deaths.

## Introduction

CVD is the leading cause of mortality worldwide.^1,2^ In 2019, 18.6 million deaths (33% of all deaths) were due to CVD,^3^ with a projection of 24 million annual deaths by 2030.^4^ In England and Wales, CVD was responsible for almost a quarter of all deaths in 2019.^5,6^ Premature mortality from CVD in England has also been attributed to greater socioeconomic inequalities in people aged <75 years.^7^

In patients at high risk of strokes, heart attacks, deep vein thrombosis, or pulmonary embolism, anticoagulation is one possible prophylactic intervention.^8^ Anticoagulants target different points of the coagulation cascade, helping to prevent blood-clot formation and the adverse effects of excessive clotting. In English primary care, the prescribing of anticoagulants increased from 15 million to 33 million doses between January 2014 and August 2019.^9^ Three main types of anticoagulants are outlined in guidance published by the National Institute for Health and Care Excellence (NICE): low molecular weight heparin (for example, enoxaparin), vitamin K antagonists (for example, warfarin), and direct-acting oral anticoagulants (DOACs; for example, rivaroxaban). The effectiveness of anticoagulants for CVD is well established. For example, adjusted-dose warfarin reduced stroke by 62% (95% confidence interval [CI] = 48% to 72%) in patients with atrial fibrillation.^10^ However, the narrow therapeutic index and frequent laboratory monitoring needed with warfarin administration have led to the development of DOACs.^11^ Bleeding associated with warfarin therapy is among the top three adverse drug reactions that cause hospital admissions in England.^12^

Coroners’ reports, previously named Rule 43 and now called Prevention of Future Death reports (PFDs), are written when the coroner believes that action is necessary to prevent a death.^13^ PFDs are sent to specific individuals or organisations, who, under regulation 28 and 29 of the Coroners (Investigations) Regulations 2013, have a duty to respond within 56 days of the date of report.^14^ Previous analysis of coroners’ reports has shown that anticoagulants were the drugs most commonly reported to have been involved in fatal medication errors in England and Wales.^15,16^ This analysis also found that coroners most commonly raised concerns regarding adverse drug reactions to prescribed medicines, followed by omissions of necessary treatment and monitoring failures. However, the authors examined only a proportion (*n* = 500) of all published coroners’ reports. Building on previous research,^15,16^ the present study aimed to assess all available PFDs between 2013 and 2019 for deaths that involved individuals with CVD, in whom the use or lack of use of anticoagulants caused or contributed to death. The authors sought to discover the following: 1) what concerns were highlighted by coroners; 2) to which individuals or organisations PFDs were addressed; and 3) whether responses were made by the individuals or organisations to whom the PFDs were sent.

## Method

A systematic case series was designed and the study protocol was preregistered on an open repository.^17^ The STROBE reporting guideline was used to write the manuscript.

### Data collection

PFDs are openly available on the Courts and Tribunals Judiciary website.^18^ Web scraping was used to automatically collect PFDs, and from the output the Preventable Deaths Database and the Preventable Deaths Tracker were created: https://preventabledeathstracker.net. The code to create the scraper is openly available on GitHub^19^ and has been previously described.^20^ The Preventable Deaths Database contains the following: the case reference number; the date of the report; the name of the deceased; the coroner’s name; the coroner’s jurisdiction; the category of death (defined by the Chief Coroner's Office); to whom the report was sent; and the URL to the Courts and Tribunals Judiciary website. For population data on deaths from CVD, the most recent (2001–2019) dataset was used of deaths registered in England and Wales, released by the Office for National Statistics (ONS) in 2020.^5^

### Eligibility of cases

All cases (*n* = 3037) in the Preventable Deaths Database were examined from July 2013 (the first date on which they were uploaded to the Courts and Tribunals Judiciary website) to December 2019. The cases were screened independently in duplicate by two authors to determine whether CVD caused or contributed to the death using a predefined algorithm (see Supplementary Figure S1). Chapter IX (Diseases of the Circulatory System) of the International Statistical Classification of Diseases and Related Health Problems 10th Revision (ICD-10) was used to align with the ONS classifications of death.^6^ When the deceased suffered from a single condition listed under chapter IX that could not be unequivocally attributed to external causes, the case was included. The authors then screened for cases where ≥1 anticoagulant caused or contributed to the death, or where the coroner suggested that had an anticoagulant been given, it would have prevented the death. Anticoagulants were defined as agents targeting different points of the coagulation pathway to prevent clot formation. Discrepancies were resolved by consensus discussion with a third author.

### Data extraction

For included cases, one author manually extracted the following variables into a predesigned Google Sheet, which was cross-examined by another author: the individuals or organisations to whom reports were sent, who responded, the due date of response and the date received; date of death; the dates on which the inquest started and ended; age; sex; setting or location of death; medical cause(s) of death; the coroner’s conclusion(s) of the inquest; relevant medical, mental health, and social history; whether any substance(s) were implicated in the death and the type of substance(s); coroner’s concern(s); and actions proposed by the coroner.

### Data analysis

To determine the annual deaths from CVD in England and Wales, the ONS data^5^ were filtered for deaths caused by conditions listed under chapter IX of ICD-10 (ICD-10 codes: I00–I99).^6^ The authors summed the number of reports written each year and compared the totals with ONS mortality data for CVD. Descriptive statistics were used to report the quantitative findings and content analysis^21^ was performed to classify the concerns and actions raised by coroners with categories derived inductively. A response rate was calculated for each organisation as the proportion of reports to which a response was submitted over the total number received. Responses were further classified as either on time (delivered within 56 days), late (submitted after 56 days), or overdue (when no response was found on the Courts and Tribunals Judiciary website). A response rate of 100% meant that an individual or organisation adhered to regulation 28 of the Coroners (Investigations) Regulations 2013 and responded to all PFDs issued by coroners.

### Software and data sharing

Python (version 3.7) was used in Jupyter Notebooks with pandas, seaborn, and matplotlib libraries to analyse the data and create figures. The data, statistical code, and study materials are openly available via the Open Science Frame^22^ and GitHub.^23^

## Results

**Figure 1.**
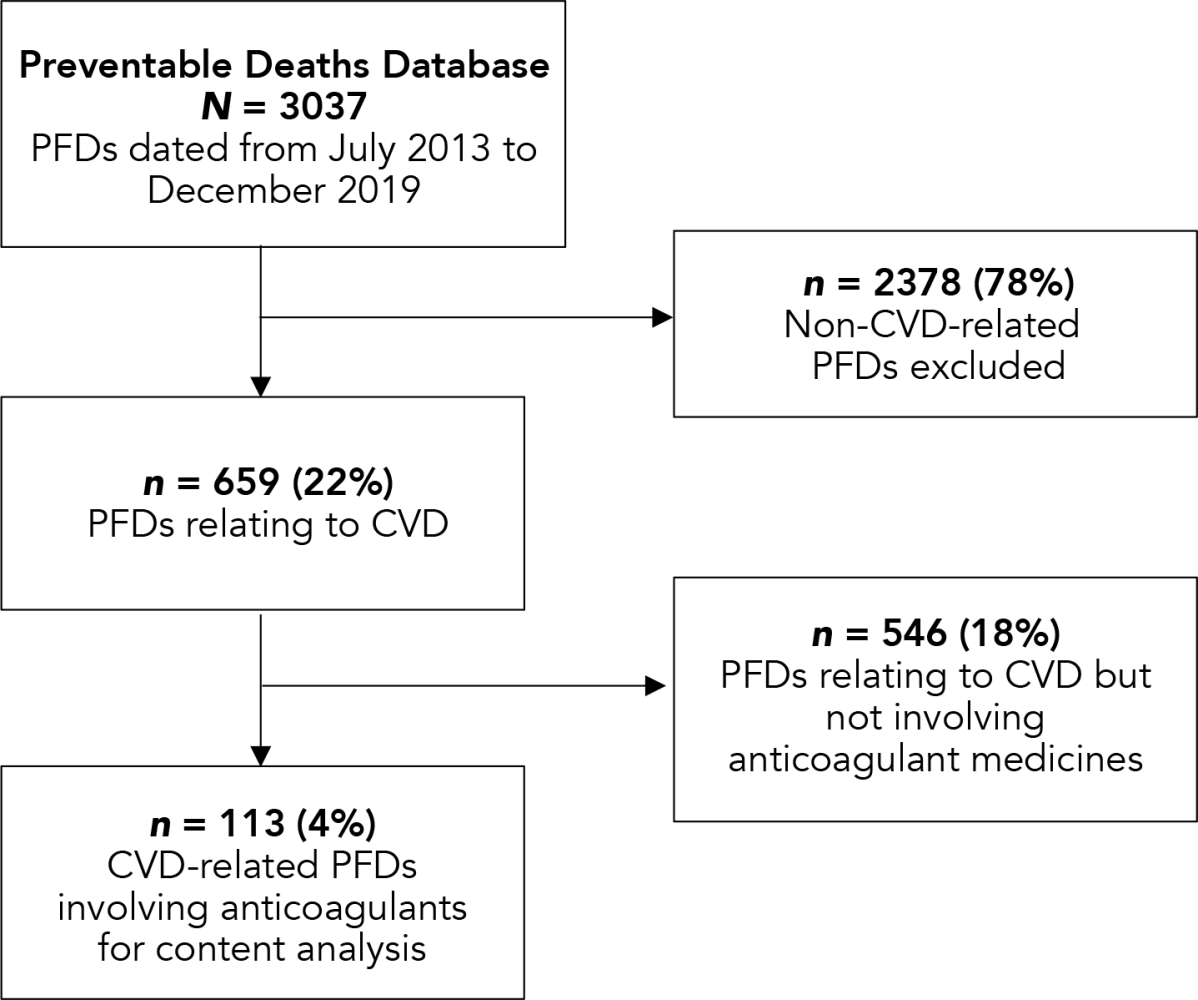
Flow diagram showing the numbers of Prevent Future Death reports (PFDs) included and excluded from the Preventable Deaths Database using the eligibility criteria for this study. CVD = cardiovascular disease.

In 659 cases (22% of all PFDs) CVD caused or contributed to the death (Figure 1). Of the CVD-related PFDs, 17% (*n* = 113) involved or mentioned the use or lack of an anticoagulant. Over the 7-year study period, there was a median of 16 (interquartile range [IQR] 15–17) CVD-related anticoagulant PFDs each year (see Supplementary Table S1).

**Figure 2.**
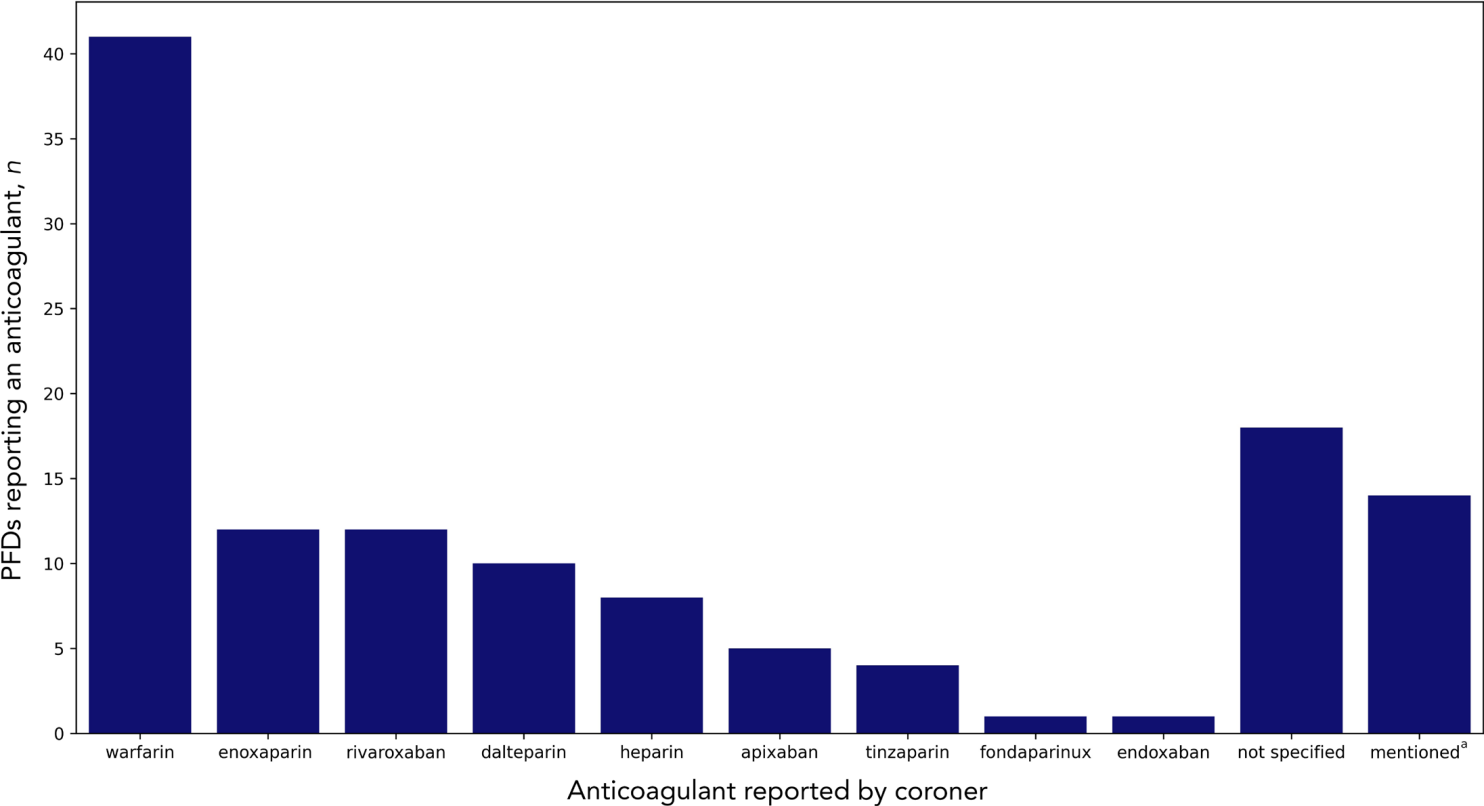
Anticoagulants reported in cardiovascular disease-related Prevent Future Death reports (PFDs). ^a^In these PFDs, the coroner suggested that if an anticoagulant had been used the death might have been prevented.

In 99 cases (88%), the use of ≥1 anticoagulant caused or contributed to the death. In 14 cases (12%), the coroner mentioned that the administration of an anticoagulant might have prevented the death (Figure 2). Warfarin (36%, *n* = 41) was the most common anticoagulant specified, followed by enoxaparin (11%, *n* = 12) and rivaroxaban (11%, *n* = 12). There were equal proportions of males (*n* = 56) and females (*n* = 57) in the 113 cases. The median age of the deceased was 76 years (IQR 61–84 years, *n* = 77).

**Figure 3.**
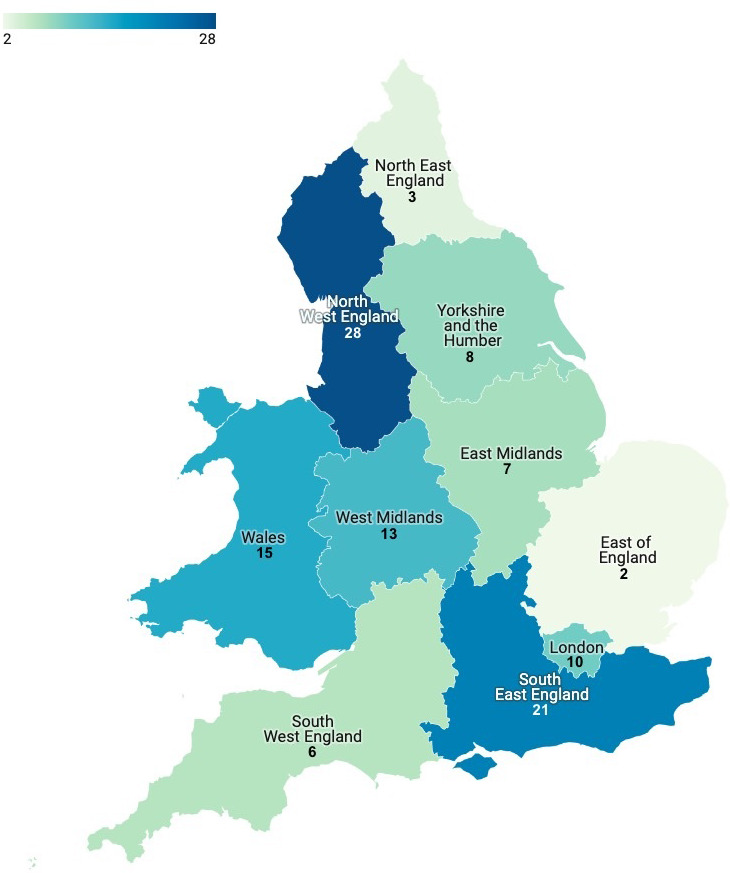
Map of where the 113 Prevention of Future Deaths reports involving cardiovascular disease that mention anticoagulation were issued by coroners in England and Wales between July 2013 and December 2019

Seventy-five coroners across 36 jurisdictions wrote 113 PFDs. Coroners in the North West (25%) and South East (19%) of England wrote the most, whereas those in the East (2%) and North East (3%) of England wrote very few (Supplementary Table S2 and Figure 3).

A total of 335 individual concerns raised by coroners were identified in the 113 cases. Using content analysis, these concerns were categorised into 51 groups and five higher-order themes, including communication, failure to follow protocols, education and training, resources, and safety (Table 1). The most common concerns were poor systems (31%), poor communication (25%), failure to keep accurate medical records (25%), and failures or delays in having appropriate assessments done (17%). Concerns most frequently belonged to the theme of following protocols (36%), followed by communication (22%), and safety issues (21%).

**Table 1.** Concerns raised by coroners, grouped by five higher-order themes, and how often they were reported

**Theme (% of concerns**)	**Concern**	**Cases, *n* (%**)
Failure to follow protocols (36)	Failure or delay in appropriate assessments	19 (17)
	Failure to monitor treatment	15 (13)
	Omission of necessary treatment	13 (12)
	Delayed treatment	12 (11)
	Failure to review medicines	11 (10)
	Failure to take a history or see the patient	10 (9)
	Failure or delay in performing necessary scans	8 (7)
	Failure to triage patients appropriately	7 (6)
	Failure to follow recommended practices	6 (5)
	Failure to follow a protocol	6 (5)
	Failure to review medical records	4 (4)
	Failure to implement national guidelines	3 (3)
	Administration of drug in error	1 (1)
	Failure to arrange supervision	1 (1)
	Inability to care for both a patient’s physical and mental health	1 (1)
	Management of medication for care home residents	1 (1)
	Medication administered despite known allergy	1 (1)
Communication (22)	Poor communication	28 (25)
	Failure to keep accurate medical records	28 (25)
	Failure to escalate deterioration in patient to the relevant medical professionals	5 (4)
	Failure to seek specialist advice when indicated	4 (4)
	Failure to follow the advice of a senior clinician	2 (2)
	Failure to warn of the consequences of not taking medication	2 (2)
	Failure to inform the patient about a medical procedure and aftercare	2 (2)
	Failure to warn of adverse drug reactions	1 (1)
	Failure to obtain informed consent	1 (1)
Safety (21)	Poor systems	35 (31)
	Discharge process	16 (14)
	Non-robust investigation following the death	15 (13)
	Safety of facilities	2 (2)
	Nature of inspections of care homes	1 (1)
	Failure to address measures identified in risk assessment	1 (1)
	Failure to make a reasonable effort to ensure patient adherence	1 (1)
Education and training (14)	Inadequate training	12 (11)
	Inappropriate dosage for the patient	9 (8)
	Failure to appreciate the risk (of giving or not giving a drug)	6 (5)
	Lack of clinical knowledge	5 (4)
	Poor clinical decision making	5 (4)
	Poor awareness of symptoms	4 (4)
	Poor awareness of rare ADRs	2 (2)
	Poor awareness of rare complications of medical procedures	1 (1)
	Failure of training	1 (1)
	Drug awareness	1 (1)
	Poor awareness of drug—drug interactions	1 (1)
	Wrong method of administration	1 (1)
Resources (7)	Absence of national guidelines	10 (9)
	Understaffing	7 (6)
	Shortage or lack of availability of appropriate medical equipment	3 (3)
	Hospital opening times or availability	2 (2)
	Unavailable drug	1 (1)
	Inability to deliver care	1 (1)

ADR = adverse drug reaction.

In 82% of CVD-related PFDs involving anticoagulants, coroners stated that 'action should be taken'. When coroners suggested further actions, their actions were grouped into 28 categories (see Supplementary Table S3).

For the 113 CVD-related PFDs involving anticoagulants, coroners sent 181 reports to 37 individuals and organisations (Table 2). Local services, such as NHS trusts (29%), hospitals (10%), and general practices (8%) addressed PFDs most frequently. By statute addressees must respond to the coroner within 56 days, but only 29% responded on time; 11% responded late and 60% were overdue. Medical societies (0%) and royal colleges (0%) had the lowest response rates, while NHS entities had the highest, albeit with half of their responses overdue. Ranking recipients by response rate and response time, NHS 111, NHS Wales, and clinical commissioning groups (CCGs) performed best (see Supplementary Table S4).

**Table 2.** Recipients of coroners' Prevent Future Death reports (PFDs) and their response rates to reports

**Addressee**	**Reports received, *n***	**Responses, *n***	**Response rate, %**	**Classification of responses, %**	
				**On time**	**Late**	**Overdue**
**NHS entities**	**118**	**60**	**51**	**38**	**13**	**49**
CCGs	3	3	100	67	33	0
NHS 111	1	1	100	100	0	0
NHS Wales	1	1	100	100	0	0
Ambulance services	7	5	71	57	14	29
NHS trusts	53	32	60	45	15	40
NHS England	4	2	50	50	0	50
Hospitals	19	7	37	26	11	63
General practices	14	5	36	29	7	64
Local health boards	3	1	33	0	33	67
University health boards	10	3	30	20	10	70
Mental health trusts	2	0	0	0	0	100
NHS Pathways	1	0	0	0	0	100
**Government**	**17**	**5**	**29**	**18**	**12**	**71**
DHSC	7	3	43	14	29	57
Local authorities	3	1	33	33	0	67
Welsh Government	7	1	14	14	0	86
**Professional bodies**	**21**	**4**	**19**	**10**	**10**	**81**
CQC	6	2	33	17	17	67
NICE	8	2	25	13	13	75
GMC	2	0	0	0	0	100
BMA	1	0	0	0	0	100
GDC	1	0	0	0	0	100
MHRA	1	0	0	0	0	100
AACE	1	0	0	0	0	100
The Renal Association	1	0	0	0	0	100
**Others**	**18**	**3**	**17**	**11**	**6**	**83**
Police	1	1	100	0	100	0
Private companies	4	1	25	25	0	75
Care homes	9	1	11	11	0	89
Carewatch	1	0	0	0	0	100
Highway maintenance	1	0	0	0	0	100
Housing associations	1	0	0	0	0	100
Local charities	1	0	0	0	0	100
**Medical societies**	**4**	**0**	**0**	**0**	**0**	**100**
BCS	1	0	0	0	0	100
BRS	1	0	0	0	0	100
ICS	1	0	0	0	0	100
RPS	1	0	0	0	0	100
**Medical royal colleges**	**3**	**0**	**0**	**0**	**0**	**100**
RCGP	1	0	0	0	0	100
RCOG	1	0	0	0	0	100
RCP	1	0	0	0	0	100
**Total**	181	72	40	29	11	60

AACE = Association of Ambulance Chief Executives. BCS = British Cardiovascular Society. BMA = British Medical Association. BRS = British Renal Society. CCG = clinical commissioning group. CQC = Care Quality Commission. DHSC = Department of Health and Social Care. GDC = General Dental Council. GMC = General Medical Council. ICS = Intensive Care Society. MHRA = Medicines and Healthcare products Regulatory Agency. NICE = National Institute for Health and Care Excellence. RCGP = Royal College of General Practitioners. RCOG = Royal College of Obstetricians and Gynaecologists. RCP = Royal College of Physicians. RPS = Royal Pharmaceutical Society.

## Discussion

### Summary

The study identified 113 premature deaths from CVD involving anticoagulants. In 88% of cases, the use of ≥1 anticoagulant resulted in death. In 12% of cases, the administration of an anticoagulant might have prevented death. The study found wide geographical variation in the issuing of PFDs and the type of information reported, with coroners in Greater Manchester writing the most. Coroners raised hundreds of concerns, some relating directly to the risks of anticoagulation and the caution with which patients on these drugs should be managed, although rarely were the concerns not already mentioned in guidance available to healthcare professionals. Most of these concerns were addressed locally and under regulation 28; 109 individuals or organisations were overdue in their response to coroners.

### Strengths and limitations

A reproducible data-collection method was used to examine all available PFDs from inception to 2019, which provided an estimated 40-fold time-saving^20^ and reduced the potential for selection bias. However, the PFDs included in the study depend on the working practices of coroners and the Chief Coroner’s Office in uploading PFDs and their responses to the website. The 113 PFDs cannot therefore represent all preventable deaths from CVD involving anticoagulants in England and Wales. There were also missing data; for example, 32% of PFDs did not report age and 16% did not specify the type of anticoagulant; this may be attributed to lack of PFD training provided to coroners. The findings are also limited by the available data and information provided by coroners in PFDs, thus it is not possible to examine the relationship between CVD and anticoagulants in causing death, or to differentiate between the appropriate use, misuse, and underuse of anticoagulants.

### Comparison with existing literature

The study builds on prior research that evaluated smaller samples of coroners’ reports.^15,16,24–26^ Compared with previous studies,^25,27^ a sex imbalance was not identified. However, it was found that new hazards were rarely identified and that most PFDs were addressed locally, as shown by Ferner *et al*,^15^ which means that valuable lessons were not widely disseminated and may be why it was found that coroners repeatedly expressed similar concerns, in line with former research.^24^ This questions whether PFDs are fulfilling their purpose. Similar to Fox and Jacobson,^25^ the present study found pronounced geographical variation in the issuing of PFDs and poor response rates. A review of the coronial system in England and Wales highlighted a lack of accountability, leadership, and quality assurance.^28^ Since there is no system in place for enforcing or auditing compliance with regulation 28 or assessing the quality of PFDs and the adequacy of responses and actions taken to prevent deaths, the findings show that the system has scope for improvement.

General practices were sent the third highest number of CVD-related PFDs involving anticoagulants, but collectively only 36% responded. This may be because of a lack of awareness of the statutory requirements and medico-legal training of GPs, as identified by previous research.^29^ During the COVID-19 pandemic, GPs called for deaths of colleagues to be reported to the coroner and PFDs to be issued.^30^ Fortunately, the value of PFDs as a tool for improving clinical practice is being recognised, and efforts are underway to widely disseminate their lessons to healthcare professionals, policymakers, and the public.^31–36^

### Implications for research and practice

Concerns raised by coroners provide lessons for prescribers and policymakers on the safety and proper use of therapies. During the COVID-19 pandemic, patients were switched from warfarin to other oral anticoagulants, given the need for less frequent blood testing.^37^ Drugs such as andexanet alfa could prove critical in the outcome of patients with severe bleeding during treatment with apixaban or rivaroxaban. Since the coroner expressed concerns (case: 2018–0032),^38^ NICE conducted an appraisal of andexanet alfa for reversing anticoagulation^39^ and published guidance on 12 May 2021.^40^ There has also been a phased launch of andexanet alfa in UK hospitals.^41^ However, it is unclear whether this was a direct result of the PFD, since no response from the Medicines and Healthcare products Regulatory Agency was published on the Courts and Tribunals Judiciary website.

When PFDs are addressed to the appropriate recipients at the national level, their actions can help prevent deaths. For example, a PFD was sent to NICE when the deceased suffered a fall while taking an anticoagulant without having the appropriate neuroimaging performed (case: 2019–0106).^42^ The coroner’s concerns were acknowledged in a NICE surveillance report^43^ and resulted in updating of the NICE guideline on head injury, to emphasise that people taking DOACs should be investigated with the same care as those taking warfarin.^44^ However, unaddressed concerns were also identified about national guidelines; for example, three-quarters of PFDs sent to NICE have no responses listed on the Courts and Tribunals Judiciary website. Restarting warfarin after a head injury is particularly important, as delays could leave the patient at risk of a stroke, but resuming too soon may lead to haemorrhage. A retrospective review of the medical charts of 256 patients admitted to a trauma centre in west Texas between 2009 and 2012 showed that patients who resumed anticoagulant therapy at 7–10 days after the injury had the best prognosis.^45^ PFDs can therefore be used to update guidance and inform future prospective cohort studies.

This study provides a database and resource (https://preventabledeathstracker.net) for future evaluations of PFDs. Further content analysis should be used to assess the 181 responses to the 113 PFDs, to assess the adequacy of actions proposed to prevent deaths and their implementation. Future research could use the open data to examine coroners' concerns in the 546 CVD-related PFDs not involving anticoagulants and their responses. In building the web scraper to collect PFDs, various inconsistencies and omissions were found on the Courts and Tribunals Judiciary website, which should be addressed to improve the quality of data. The missing data from PFDs that have been highlighted reveal target areas for coronial training in the writing of PFDs.

In conclusion, this study used sophisticated, reproducible, and internationally recognised data-collection methods^20^ to demonstrate that PFDs provide valuable lessons when prescribing anticoagulants and managing patients with CVD. However, it is unclear whether actions are being taken to incorporate such lessons. To improve patient safety, lessons should be widely disseminated and used in practice.

## Data availability:

Study materials, protocol, data, and statistical code are openly available on the OSF (https://osf.io/qkfjp/) and GitHub (https://github.com/georgiarichards/CVD_anticoags).
